# New York Letter

**Published:** 1879-12

**Authors:** 


### Domestic Correspondence.

### ARTICLE X.

*Editors* CHICAGO MEDICAL JOURNAL AND EXAMINER:

Quite a number of New York medical men spent their summer vacations abroad, taking advantage in this way of the several medical congresses which met at Cork, Amsterdam, and Montpellier. No one made a more triumphal progress, or came back with more honors than did Dr. Sayre. With his plaster of Paris bandages, his suspension apparatus, and his son Charley, he straightened out the crooked, raised the bed-ridden, and captured all his audiences. There was a little coldness, to be sure, amongst Parisian surgeons; and an English medical man wrote something, based upon a hundred cases, that was scarcely favorable to our countryman or his jacket. This, however, was nothing compared to the overwhelming tide of enthusiasm which met the eminent doctor almost everywhere. Dr. Sayre is certainly a remarkable man, and has already had a distinguished career. With study, common sonse, and an impetuous energy in asserting his beliefs, he has ridden to fame upon one pathological idea and two therapeutical principles. The supremacy of traumatism over scrofula, the necessity of rest, and the importance of extension, are the ideas which have controlled his surgical opinions and methods, and by the vehement utterance of which he won his reputation. There have been, of course, certain minor things, such as reflex action and the prepuce, which have broadened as it were, his views, and ornamented with lighter touches his career. The qualities of his character are strongly marked and easily seen. He has excellent judgment, a practical rather than studious mind, and much mechanical skill. His quick appreciation of what is valuable in therapeutical appliances, is much greater than his own inventiveness. He appropriates more than he originates (unless we except his style of expression). But he champions what is good, and generally proves to others his correctness. Dr. Sayre's reliability has sometimes been called in question. His success is said to be too uniformly remarkable.

There is probably no doubt that in the eloquence of his clinical expositions, or in the heat of his spirited objurgations, he sometimes wanders from the hackneyed limits of the actual (I hope I have put the thing mildly enough). The true story of clinical cases is often embarrassing to any clinical teacher who wishes to present a disease in all its classical and rotund completeness. And if a man receives from doting mothers three letters full of gratitude and terms of effusive endearment, he is apt to think that there have been six. Cases whose hospital record is "discharged cured," will come back sometimes with a terrible limp and pain in the hip. It is rumored, indeed, in the orthopedic wards of Bellevue Hospital, that hip-joint disease never is cured with any satisfactory result; and this expresses a considerable amount of truth.

However, inaccuracies of statement often imply only a superabundant enthusiasm and have no further moral significance. No one can deny that Dr. Sayre has done work which justly entitles him to the highest honors of the profession as well as the gratitude of his fellow men. I should not omit to mention that no one, however poor, is ever turned from his office and that the amount of private charity thus dispensed is very great indeed.

The New York medical colleges have opened with their usual quota of students. Bellevue Hospital Medical College, as is now well known, has announced a change in its course. After the present session it will be graded; annual examinations and three years attendance upon lectures will be required.

It requires much courage for a school like that of Bellevue to make such a change, and great credit is due the faculty, both on this account and because its example is so conspicuous that the other city colleges cannot avoid eventually following it. There is a great cry just now for higher medical education and the colleges are beginning to show that they feel it. It is absurd to expect that every medical graduate shall be a gentleman of wide accomplishments and elegant culture, but there are just now two evils which we have to face: a great surplus of young graduates and a great deficiency of education both general and professional amongst them. Medical colleges should be made to remember this and shape their course accordingly. As they will only do it partially, however, we must rely on the law of supply and demand to settle the question eventually.

At a meeting of the County Medical Society last spring, a paper on laryngeal phthisis was read by Dr. Bosworth, in which he announced the possibility of its cure. In the discussion of the subject afterwards, a number of prominent laryngologists took part, and turned the question finally to the propriety of performing tracheotomy in the disease. No one was positively in its favor, but a number of cases tending to show that it was justifiable and even imperative were quoted. Since then several other cases have been brought to my notice. One of these, operated on by Dr. Oppenheimer, of the New York Eye and Ear Infirmary, seemed to show the operation to be absolutely curative in its effect. Two others gave much relief to the patients. From cases of which I have had a more personal knowledge, however, it has seemed to me that the good results of this operation are much exaggerated, and I believe that the growing feeling in its favor is a dangerous one. It is liable to give the patient a shock from which he does not rally. It introduces air directly, unsifted and cold, upon bronchial mucous membrane. It gives the patient a great deal of annoyance, and is generally thought to be a terrible thing by the friends. But more than all, it makes expectoration difficult, and the muco-pus accumulates in the cavities of the lungs, to the discomfort and injury of the patient. I don't believe that the operation should ever be done, unless the indication to secure euthanasia is very strong.

You have, I hear, a very active health board in Chicago. Ours of this city has been boasting of what it has accomplished, during the present summer, in the way of saving children's lives. By its system of tenement-house inspection, by sea-side and country sanitaria, by water excursions and land excursions, a great deal of fresh air during the summer has been introduced into the lungs and system of the rising generation. In 1875, the deaths from diarrhœal diseases, mainly amongst children, were 2,997; in 1876 they were 3,060; in 1877, when sanitary measures began, they were 2,657; in 1878, about 2,052; and in 1879, about 2,084. Thus there has been a decrease of nearly a thousand deaths amongst the children, with a corresponding diminution in the sickness rate. So much for sanitary science. It should be remembered, however, that a great deal of this is due to private charity, and that the health board was only one of the agents. Just now, sanitary science and State medicine are "booming," and enthusiastic men are beginning to give us to understand that if they will only be allowed full swing, sickness will be abolished and society become immortal. This sounds very nice, but it will be terrible for the doctors. We can encourage hygiene until it has reduced the mortality to fourteen or fifteen per thousand. The medical profession must then join anti-sewerage and anti-vaccination societies, and become the deadly enemy of sanitary progress. What would medicine be without typhoid-fever, or if the *bacillus malariæ* were forever exterminated? C. L. D.

